# Trends in obesity prevalence among Brazilian adults from 2002 to 2013 by educational level

**DOI:** 10.1186/s12889-019-7289-9

**Published:** 2019-07-18

**Authors:** Danilo Cosme Klein Gomes, Rosely Sichieri, Eliseu Verly Junior, Cristiano Siqueira Boccolini, Amanda de Moura Souza, Diana Barbosa Cunha

**Affiliations:** 1 Institute of Social Medicine, State University of Rio de Janeiro, São Francisco Xavier, 524 – 7 andar, Bloco E, sala E-7017B, Maracanã, CEP, Rio de Janeiro, RJ 20550-900 Brazil; 2 Institute of Scientific and Technological Communication and Information in Health, Oswaldo Cruz Foundation, Rio de Janeiro, RJ Brazil; 3 Institute for Studies in Collective Health, Federal University of Rio de Janeiro, Rio de Janeiro, RJ Brazil

**Keywords:** Obesity, Overweight, Body mass index, Prevalence, Educational status

## Abstract

**Background:**

Obesity ranks as a major public health problem in many countries in the world. The obesity-socioeconomic status relationship is not well established in middle-income countries.

**Methods:**

The aim of this study was to estimate the obesity and overweight trends from 2002 to 2013 by sex, age, and educational levels among Brazilian adults. The panel prevalence trend study was conducted, considering the sample weights and study design. Three nationwide surveys were analyzed: the Household Budget Survey 2002/2003 and 2008/2009, and the National Health Survey 2013. The total sample was 234,791 adults aged 20–59 years.

**Results:**

The prevalence of obesity increased from 7.5 to 17.0% from 2002 to 2013 among adults aged 20–39 years and from 14.7 to 25.7% among those aged 40–59 years, slightly higher among young women. In each survey, education was positively associated with the prevalence of obesity among men, whereas this association was negative among women. The greatest increase in the prevalence of obesity was 90% (11.9 to 22.5%) and occurred from 2008 to 2013 among women with secondary educational level, whereas at the pre-primary level there was a 42% (20.4 to 29.0%) increase.

**Conclusions:**

Obesity prevalence in Brazil continued to increase, mostly among women with secondary education. Policies aimed at reducing the prevalence of obesity should consider sociodemographic characteristics in the population.

## Background

Obesity is a risk factor for many chronic noncommunicable diseases and poses a great challenge to global and Brazilian health systems. More than 74% of all deaths in Brazil are attributed to chronic noncommunicable diseases [1], with less privileged groups bearing a large share of this burden [2]. From 1980 to 2010, the prevalence of diabetes in Brazil increased from 7.4 to 15.7% [3].

The worldwide prevalence of obesity has nearly tripled since 1975. In 2016, 39% of adults worldwide were overweight, and 13% were obese [4]. Although obesity is a multifactorial disease, environments that promote a higher energy intake have greatly influenced this epidemic [5].

Low and middle-income countries showed the greatest increase in the prevalence of obesity between 1975 and 2014, and will likely soon exceed the prevalence in developed countries. In 2014, Brazil, a middle-income country, was ranked third in the world for the absolute number of obese adult men (11.9 million), falling behind only China and the United States. The country also occupied the fifth position for the number of obese women (18.0 million) [6]. Also, socioeconomic status (SES) and obesity are positively associated in lower-income countries but negatively associated in higher-income countries [7, 8]. However, the obesity-SES relationship is not well established in middle-income countries [9] where the prevalence may reach those of developing countries in the coming years [10]. In Brazil, telephone surveillance surveys showed an increase in the prevalence of obesity from 2006 to 2017, reaching 18.9% of adults in the last survey; that survey also showed an increase in the prevalence of obesity among women with lower educational level [11]. These telephone surveys in Brazil were limited by three factors. Only capitals were included, there was a lack of telephones in houses of the poorest population, and weight and height were self-reported. This was not the case in the national surveys included in the present study. Also, educational levels in Brazil showed greater improvement from 2002 to 2013. Secondary levels of education almost doubled in this period [12].

The identification of obesity trends in subgroups of the nationwide population may help to better plan specific prevention measures. Therefore, the purpose of this study was to analyze overweight and obesity trends according to sex, age, and educational levels in Brazilian adults.

## Methods

Data from three nationally representative surveys were analyzed: the Household Budget Survey–Pesquisa de Orçamento Familiar (POF) conducted in 2002/2003 and 2008/2009, and the National Health Survey–Pesquisa Nacional de Saúde (PNS), undertaken in 2013. The Brazilian Institute of Geography and Statistics (IBGE) was responsible for the surveys. The raw data and questionnaires for data collection are publicly available [13–15].

The sampling procedures included multi-stage stratified clusters. In the first stage of the POFs, primary sampling units (PSUs) were selected by systematic sampling proportionally to the number of households. For the second stage, households were selected by simple random sampling without replacement. Anthropometric measurements were taken from all individuals present in the selected households at the time of the interview.

The PNS sample was a subsample of the Master Sample of the Integrated Household Surveys System of the Brazilian Institute of Geography and Statistics. Cluster sampling was performed in three stages: the PSUs comprised sectors of the census and were obtained by simple random sampling among those previously selected for the Master Sample. It maintained the PSU stratification used in the Master Sample. The secondary unit included 10 to 14 households selected from each PSU, and the third unit included one person aged 18 years or more from each household who responded to the individual component of the questionnaire distributed by the PNS.

The initial databases included 182,333, 190,159, and 205,546 individuals from the 2002/2003, 2008/2009, and 2013 surveys, respectively. This study analyzed data from adults between 20 and 59 years of age of both sexes. Pregnant women were excluded, resulting in a sample comprising 234,791 adults: 89,651 (2002/2003), 100,956 (2008/2009) and 44,184 (2013). Weight and height were measured similarly in all three surveys using portable digital scales and wall-mounted stadiometers. Trained interviewers performed all anthropometric measurements and individuals were asked to remove their shoes before measurements. The classification of nutritional status was based on body mass index (BMI). Overweight and obesity were defined as BMI between 25.0–29.9 kg/m^2^ and greater than 30 kg/m^2^, respectively [16].

Data regarding education were collected in the POFs as years of education and as levels of education (in seven categories) in the PNS. In order to allow comparability between the surveys, harmonization was carried out following the International Standard Classification of Education: pre-primary (0 to 7 years of study or incomplete primary school), primary (8 to 10 years of study, complete primary school or incomplete secondary school), secondary (11 to 14 years of study, complete secondary school or incomplete tertiary school) and tertiary (greater than 15 years of study, complete tertiary school or more) [17].

The prevalence (percentage) of overweight and obesity were estimated by sex, age group (20–39 and 40–59 years), and educational level (pre-primary, primary, secondary and tertiary). Logistic regression was used to evaluate trends in the prevalence of obesity and overweight. To account for the gaps between the surveys, we encoded the 2002/2003 survey as 2, the 2008/2009 survey as 8, and the 2013 survey as 13 and used the 2002/2003 survey as the reference. All analyses were stratified by sex. Interactions of age group, educational level, and survey were assessed in order to test the effect modification in the prevalence of obesity trend by the educational level. Also, a logistic regression model was used to test the possibility of a cohort effect, including a cohort indicator variable with 4 categories (1950, 1960, 1970, and 1980). The variable outcome was obesity (yes/no) and this model was adjusted by age as a continuous variable. All analyses accounted for the sample weights and for the effect of the sample design, using SAS software version 9.4 (SAS Institute Inc., Cary, NC).

## Results

**Fig. 1:**
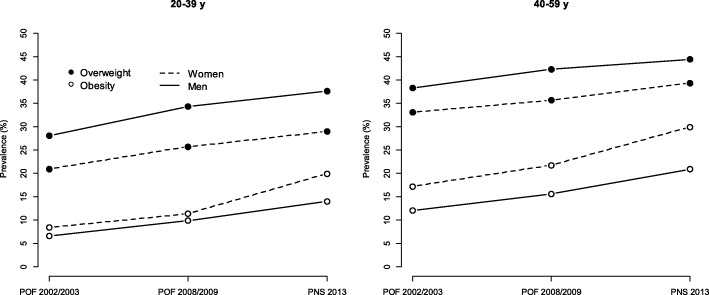
Weighted prevalence of overweight and obesity among Brazilian adults by age group and sex. Brazil, 2002–2013. Note: POF, Pesquisa de Orçamento Familiar - Household Budget Survey; PNS, National Health Survey - Pesquisa Nacional de Saúde

From 2002 to 2013, the prevalence of overweight and obesity increased in both sexes and with age in all surveys. Among women, the prevalence of obesity was highest in all surveys for both age groups. Men had a higher prevalence of overweight and a lower prevalence of obesity compared to women in all surveys (Fig. 1).

Overall, the prevalence of obesity increased from 7.5% (7.0–7.9) to 17.0% (16.2–17.8) from 2002 to 2013 in adults aged 20–39 years and from 14.7% (14.0–15.4) to 25.7% (24.5–26.9) among those aged 40–59 years. The prevalence of obesity in the 20–39 year age group from 2002 to 2013, increased from 6.6% (6.0–7.1) to 14.0% (12.8–15.2) among men and 8.4% (7.8–9.0) to 19.9% (18.7–21.1) among women; in the 40–59 year age group in the same period, the prevalence of obesity increased from 12.0% (11.1–12.9) to 20.9% (19.3–22.5) among men and from 17.2% (16.3–18.1) to 29.9% (28.3–31.5) among women (Fig. 1).

Figure 1 also indicates a cohort effect since the prevalence of obesity in the lower category of age in 2013 was higher than that in the older group at the beginning of the period, which was confirmed in the logistic regression models (*p* < 0.05).

There was an increase in the proportion of individuals with the highest educational levels in both sexes from 2002 to 2013. For both sexes, the greater changes in the educational level occurred at the tertiary level between 2002 and 2013 and at the secondary level between 2008 and 2013 (Table 1).

**Table 1:** Sample size (n) and percentage (%) by sex and educational level, weighted prevalence and 95% confidence interval of overweight and obesity among Brazilian adults according to educational level and sex. Brazil, 2002–2013

		POF 2002/2003			POF 2008/2009			PNS 2013		
Schooling		Sample	Overweight	Obesity	Sample	Overweight	Obesity	Sample	Overweight	Obesity
		n %	**Prevalence** 95% CI	**Prevalence** 95% CI	n %	**Prevalence** 95% CI	**Prevalence** 95% CI	n %	**Prevalence** 95% CI	**Prevalence** 95% CI
Men	Pre-primary	26,576	29.4	8.1	24,104	35.1	11.3	7,001	37.9	15.1
		59.3	28.4–30.4	7.5–8.8	48.7	34.2–36.0	10.6–12.0	36.1	35.9–39.9	13.6–16.6
	Primary	6,973	33.4	8.0	7,893	37.6	11.9	3,100	36.6	16.3
		15.6	31.4–35.4	7.0–9.1	16.0	35.9–39.3	10.7–13.3	16.0	34.0–39.2	13.9–18.8
	Secondary	8,429	34.4	9.0	13,848	38.6	13.0	6,770	43.4	17.2
		18.8	32.5–36.2	7.9–10.1	28.1	37.3–39.9	12.6–14.5	34.9	41.4–45.4	15.7–18.9
	Tertiary	2,846	40.2	11.8	3,571	45.5	13.0	2,529	46.8	22.1
		6.3	37.2–43.3	9.9–14.1	7.2	43.1–48.1	11.6–14.6	13.0	43.6–49.9	19.5–24.9
Women	Pre-primary	24.296	29.7	15.1	22,552	33.6	20.4	7,979	37.3	29.0
		54.2	28.7–30.7	14.3–15.9	43.8	32.6–34.5	19.6–21.2	32.2	35.4–39.3	27.3–30.8
	Primary	6,829	24.4	9.9	7,709	29.9	15.0	3,854	36.4	25.1
		15.2	22.5–26.3	8.7–11.2	15.0	28.3–31.4	13.8–16.2	15.5	33.7–39.1	22.8–27.5
	Secondary	10,092	21.1	7.5	16,243	26.9	11.9	9,209	32.0	22.5
		22.5	19.7–22.5	6.7–8.5	31.5	25.9–27.9	11.1–12.8	37.2	30.4–33.7	21.0–24.2
	Tertiary	3,610	20.2	9.5	5,036	25.4	12.2	3,742	29.4	18.5
		8.1	17.6–22.7	7.6–11.7	9.7	23.5–27.0	10.8–13.7	15.1	27.4–31.7	16.1–21.0

Note: POF, Pesquisa de Orçamento Familiar - Household Budget Survey; PNS, National Health Survey - Pesquisa Nacional de Saúde; n, number; CI, confidence interval. Age-Adjusted Prevalence

The prevalence of overweight and obesity increased over time for all educational levels and sex. In 2013, the highest prevalence of obesity was at the tertiary level among men (22.1%) and at the pre-primary level among women (29.0%), while the highest prevalence of overweight was at the tertiary level among men (46.8%) and at the pre-primary level among women (37.3%). The greater of changes in the prevalence of obesity occurred between 2008 and 2013 among women at the secondary level (11.9 to 22.5%). The highest educational levels had the highest prevalence of overweight and obesity for men and the lowest for women; this suggests a negative relationship between educational level and obesity in women and a positive one in men (Table 1).

Overall, for both age groups the odds of being obese doubled in 2013 compared to those in 2002, while the chance of being overweight increased by almost 1.5 times in both sexes in the same period. Young women had the highest chance of becoming obese (Table 2).

**Table 2:** Odds ratio (OR) and 95% confidence intervals for obesity and overweight trend among Brazilian adults compared to 2002/2003

			2008/2009		2013	
		Age	OR	95% confidence interval	OR	95% confidence interval
Obesity	Men	20–39	1.5	1.4–1.7	2.2	1.9–2.5
		40–59	1.4	1.2–1.5	1.8	1.6–2.0
	Women	20–39	1.4	1.2–1.5	2.5	2.3–2.8
		40–59	1.3	1.2–1.5	1.9	1.7–2.0
Overweight	Men	20–39	1.3	1.2–1.4	1.4	1.3–1.5
		40–59	1.2	1.0–1.3	1.2	1.0–1.3
	Women	20–39	1.3	1.2–1.4	1.4	1.3–1.5
		40–59	1.1	1.0–1.2	1.2	1.1–1.3

**Fig. 2:**
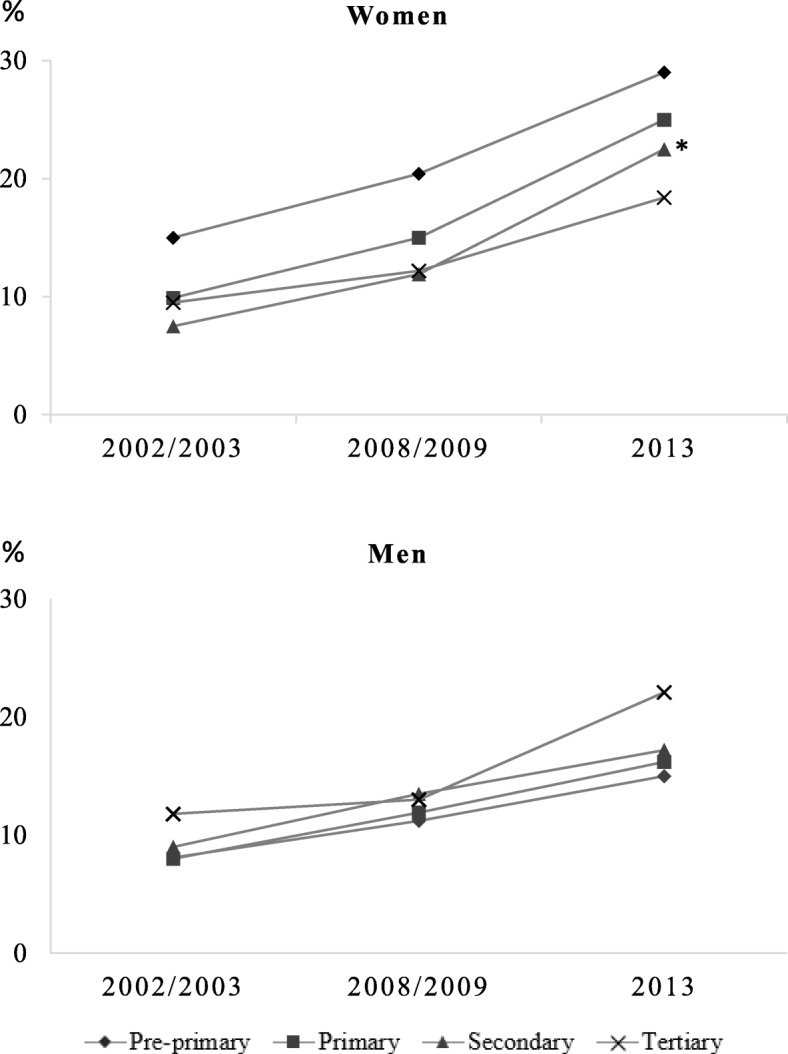
Change in prevalence of obesity among Brazilian adults by educational level and sex. Brazil, 2002–2013. Note: * p-value < 0.05 for interaction term survey-educational level

In 2002 and 2008, women with tertiary and secondary educational levels had lower prevalence of overweight and obesity (Table 1); however, from 2008 to 2013, the rate of change of the prevalence of obesity was higher among women with secondary educational level compared to those with low education, as indicated by the statistically significant effect modification of survey observed in Fig. 2. The interaction term, which measured the change in time differences among women, had *p*-values lower than 0.05 for women.

## Discussion

This study reveals a significant increase in the prevalence of overweight and obesity in the adult Brazilian population from 2002 to 2013, mainly among women. Educational level improved in this period and the proportion of people of both sexes at the highest level almost doubled from 2002 to 2013. The changes in the prevalence of overweight and obesity were not homogeneous by educational level and sex. A greater rate of change occurred among women with secondary educational level, whereas men at all educational levels had a similar increase in the prevalence of obesity.

Although obesity is a problem faced by almost all countries [7, 18, 19], the prevalence varies by sex as well as by socioeconomic level [20]. In European countries, the differences according to sex are small, whereas women in the United States, Mexico, Fiji Island, South Africa, and Brazil are at greater risk of obesity compared to men [6]. A systematic review in 2004 identified a shift towards poor women in developing countries i.e. an increase in obesity prevalence amongst poor women in developing countries [20].

In contrast with our findings, studies from high-income countries have shown a negative association between educational level and obesity in both sexes [9, 18, 21, 22], with women being more influenced by the educational level [18, 22].

Similar to our results, a 2011–2014 survey conducted in the United States showed a higher prevalence of obesity among women than among men, and an even higher prevalence of it among the less educated women [23]. In medium-income countries, a positive association between obesity rate and educational level has been observed, while this association tends to be negative in high-income countries [24]. Our data indicate that Brazil has a more complex situation: the prevalence among women is negatively associated with increased education and lower rates of obesity among those with higher educational levels, while men had higher rates of obesity with higher educational levels, similar to the poorest countries. Also, the trend over decades is changing. In a previous study conducted in Brazil, the prevalence of obesity increased among men from 1989 to 2003. The increases were greater among the poor, but the obesity rate remained stable among women [25]. The more recent population-based data used in our analysis also shows a negative association between obesity and educational level in women and a positive association in men. The movement of women from the pre-primary and primary educational level to the secondary educational level sub-population due to many successful public policies aimed at increasing education in Brazil [26, 27] is likely to explain the greater increase in the prevalence of overweight and obesity among women with a secondary level of education. Moreover, the characteristics of the women in the secondary educational group have probably changed, including more poor/rural women. However, there was a clear graded association between level of education and prevalence of overweight and obesity in both sexes. Similar to our findings, a recent Swedish study showed that the most important increase in prevalence of obesity occurred at the middle educational level [28].

We also identified a cohort effect for both sexes. The increase in energy intake and the decrease in energy expenditure over the last decades is the hypothesis for the birth cohort effect found in our study [29]. One limitation of this consideration is that the subpopulation at the secondary school level in 2013 was likely different from the subpopulation at the secondary level in 2002 i.e. poorer children are more likely to access a higher level of education in the later cohorts [30].

Our study has several strengths. The three national surveys of adults in Brazil used the same anthropometric measurements, the same resources and field worker training and similar sampling methodologies.

A possible weakness is the use of BMI instead of body fat, that may have changed across decades [31, 32]. However, in Brazil, BMI and body fat are strongly correlated [33]. Another limitation is that the questionnaire for capturing educational level was similar among POFs, but different from those used in the PNS.

The increased prevalence of overweight and obesity in the last decade among Brazilian adults observed in this study has important implications for public health due to their association with chronic medical conditions and to the high morbidity and mortality related to obesity [32] in addition to public health costs. The estimated total annual public cost associated with overweight and obesity-related diseases in Brazil was US$ 2.1 billion [34].

## Conclusions

This study shows an increase in the prevalence of obesity in the last decade among Brazilian adults of both sexes and at all levels of education. The highest educational levels had the highest prevalence of obesity and overweight for men and the lowest for women, and the increase in the prevalence of obesity among women occurred more prominently at the secondary educational levels. This may be due to changes in average levels of educational attainment throughout the study period. These findings reveal the need for nutritional education campaigns and policies to curb the obesity epidemic, since the recent educational improvements during this period coursed with cohort effect and less than the expected educational effect in the obesity prevalence.

## Data Availability

Details about each survey (data and questionnaires for data collection) can be found at https://ww2.ibge.gov.br/home/estatistica/populacao/condicaodevida/pof/2002/default.shtm for POF 2002/03, https://ww2.ibge.gov.br/home/estatistica/populacao/condicaodevida/pof/2008_2009/ for POF 2008/09 and https://ww2.ibge.gov.br/home/estatistica/populacao/pns/2013/default.shtm for PNS 2013.

## Abbreviations

BMI: Body mass index
CI: Confidence interval
IBGE: Brazilian Institute of Geography and Statistics
OR: Odds ratio
PNS: Pesquisa Nacional de Saúde - National Health Survey
POF: Pesquisa de Orçamento Familiar - Household Budget Survey
PSU: Primary sampling units
SES: Socioeconomic status
